# Fusion Method to Estimate Heart Rate from Facial Videos Based on RPPG and RBCG

**DOI:** 10.3390/s21206764

**Published:** 2021-10-12

**Authors:** Hyunwoo Lee, Ayoung Cho, Mincheol Whang

**Affiliations:** 1 Department of Emotion Engineering, Sangmyung University, Seoul 03016, Korea; lhw4846@naver.com (H.L.); joa6391@gmail.com (A.C.); 2 Department of Intelligence Informatics Engineering, Sangmyung University, Seoul 03016, Korea

**Keywords:** heart rate measurement, remote HR, remote PPG, remote BCG, blind source separation

## Abstract

Remote sensing of vital signs has been developed to improve the measurement environment by using a camera without a skin-contact sensor. The camera-based method is based on two concepts, namely color and motion. The color-based method, remote photoplethysmography (RPPG), measures the color variation of the face generated by reflectance of blood, whereas the motion-based method, remote ballistocardiography (RBCG), measures the subtle motion of the head generated by heartbeat. The main challenge of remote sensing is overcoming the noise of illumination variance and motion artifacts. The studies on remote sensing have focused on the blind source separation (BSS) method for RGB colors or motions of multiple facial points to overcome the noise. However, they have still been limited in their real-world applications. This study hypothesized that BSS-based combining of colors and the motions can improve the accuracy and feasibility of remote sensing in daily life. Thus, this study proposed a fusion method to estimate heart rate based on RPPG and RBCG by the BSS methods such as ensemble averaging (EA), principal component analysis (PCA), and independent component analysis (ICA). The proposed method was verified by comparing it with previous RPPG and RBCG from three datasets according to illumination variance and motion artifacts. The three main contributions of this study are as follows: (1) the proposed method based on RPPG and RBCG improved the remote sensing with the benefits of each measurement; (2) the proposed method was demonstrated by comparing it with previous methods; and (3) the proposed method was tested in various measurement conditions for more practical applications.

## 1. Introduction

Remote sensing of vital signs has been studied to improve the measurement burden. In particular, camera-based methods allow one to measure heart rate using a smartphone both anywhere and anytime. Despite the potential of camera-based methods, they have still been limited in their applications in daily life due to the noise generated by illumination variance and motion artifacts [1].

The camera-based method is based on two concepts, i.e., color and motion. First, the color-based method uses the principle of photoplethysmography (PPG), and thus it is called remote PPG (RPPG). RPPG measures the color variation generated by reflectance of blood due to the cardiac cycle from the heart and the head through the carotid arteries [2]. The color variance is most clearly measured at the green wavelength (i.e., 510~560 nm) since the pulsations of the arteries are able to be monitored through elastic-mechanical interaction of deep arteries with the superficial dermis [3]. However, the illumination variance of various frequency ranges that occur in daily life distorts the color variance caused by the heartbeat, so it is difficult to remove the noise using only green color. The studies on RPPG have focused on the blind source separation (BSS) method from the RGB colors to overcome the noise of illumination variance. Poh et al. [4] first proposed RPPG, which extracts the color variation from the RGB colors and estimates the plethysmographic signal by using the BSS method based on independent component analysis (ICA). Xu et al. [5] reduced the noise of illumination variance using the partial least squares (PLS) method and multivariate empirical mode decomposition (MEMD). They evaluated their method in illumination changing conditions and showed more improved accuracy than the ICA-based method. Zhang [6] applied the separation of the luminance on RPPG by converting from RGB color space to LAB color space. They demonstrated that the luminance separation is resistant to natural, motion-induced, and artificial illumination variances. Although RPPG has been improved to overcome the noise of illumination variance, it is still difficult to apply in daily life since all RGB colors are affected by lighting at the same time and most proposed noise removal methods were validated in limited scenarios.

Second, the motion-based method uses the principle of ballistocardiography (BCG), so it is called remote BCG (RBCG). The RBCG measures the subtle motion generated by the contraction of the heart and the ejection of the blood from the ventricles into the vasculature [7]. The subtle motion is sensitive to measurement since its displacement is small at about 0.5 mm [8]. Thus, the major motion such as head movement and facial expression makes the measurement of subtle motion difficult. The studies on RBCG have developed by applying the BSS method to the subtle motions extracted from as many feature points as possible. Balakrishnan et al. [8] first proposed RBCG which extracts the subtle motions from feature points of the forehead and nose regions and applied the BSS method based on principal component analysis (PCA). Shan et al. [9] extracted the subtle motion from one feature point of the forehead region and applied the ICA on the displacement calculated in both the x-axis and y-axis. Haque et al. [10] increased the number of feature points by combining good features to track (GFTT) with facial landmark detection based on the supervised descent method (SDM). They demonstrated that increasing the number of feature points overcomes the tracking noise for extracting the subtle motion. Hassan et al. [11] improved the subtle motion extraction by the skin color-based foreground segmentation and the motion artifact removal by the BSS method based on singular value decomposition (SVD). Despite the improvement of RBCG, it is difficult to apply this method in daily life due to the limitation of computational performance in real time on the measurement device and of noise removal on irregular motion artifacts.

Recent studies on remote sensing have focused on the combining of RPPG with RBCG. Shao et al. [12] developed the simultaneous monitoring of RPPG and RBCG. They demonstrated that it potentially provides a low-cost solution based on a single camera in daily life, but did not consider combining them together. Liu et al. [13] combined RPPG with BCG measured using an additional motion sensor, not a camera. They corrected the tracking noise caused by motion artifacts in RPPG by referring to BCG. They only used BCG to remove motion artifacts in RPPG and did not consider their combination. Thus, it is still necessary to develop the fusion method of RPPG and RBCG by considering their interaction effect.

In summary, the BSS method has been developed to overcome the noise of illumination variance and motion artifacts in both RPPG and RBCG. Also, the fusion method of RPPG and RBCG need to be further developed by considering their interaction effect. Thus, this study hypothesized that the BSS-based combining of RPPG and RBCG can improve the accuracy and feasibility of remote sensing in daily life. This study examined the BSS methods using ensemble averaging (EA), PCA, and ICA to estimate heart rate based on combining RPPG with RBCG. The proposed method was compared with the previous methods, which only used the color variation (i.e., RPPG) or the subtle motion (i.e., RBCG). The contributions of this study can be summarized as follows: (1) the proposed method based on RPPG and RBCG improved the remote sensing with the benefits of each measurement; (2) the proposed method was demonstrated by comparing it with previous methods; and (3) the proposed method was tested in various measurement conditions for a more practical application.

## 2. Proposed Method

**Figure 1:**
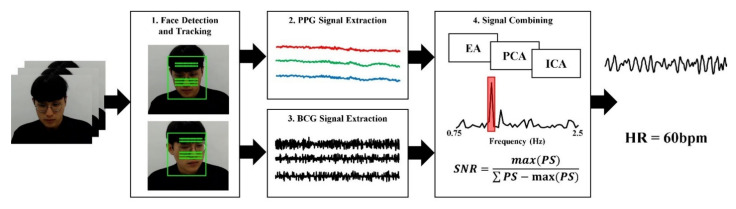
Overview of proposed method.

This study proposed a fusion method to estimate heart rate based on RPPG and RBCG by their ensemble averaging. Figure 1 depicts the procedure of the proposed method. First, the face was detected and tracked from the consequence frame of facial video. Then, photoplethysmographic and ballistocardiographic signals were extracted from the face by RPPG and RBCG, respectively. These signals were combined with each other to minimize noise and to maximize cardiac components. Finally, the heart rate was estimated from the combined signal in the frequency domain.

### 2.1. Face Detection and Tracking

To extract color variation and subtle motion, the face was detected and tracked from facial video. This study focused on low misdetection and fast inference time for practical application. The Viola-Jones algorithm [14] is a basic and widely known face detection using AdaBoost with Haar features. AdaBoost selected the candidates from an image using simple Haar features and detected the face from the candidates using complex Haar features to improve inference time. It is easy to use since it is implemented in the OpenCV library [15], but it frequently mis-detected the face. Histogram of oriented gradients (HOG) algorithm [16] is also a representative face detection algorithm implemented in the DLIB library [17]. It computed the spatial gradients from an image and detected the face using histogram of gradient orientation. Although it has less misdetection than the Viola-Jones algorithm, it can detect only the frontal face and has a slow inference time.

Recently, face detection methods using deep learning have been proposed and have shown more enhanced performance than the Viola-Jones and HOG algorithms. Although their algorithm was complex and difficult to implement, they are easier to use than before by development of open-source framework such as Tensorflow [18]. Thus, this study employed the single shot detector (SSD) [19] with ResNet [20] trained by WIDER FACE dataset [21]. The ResNet extracted a high dimensional feature map which has facial features such as contrast or facial contour from an image. The SSD detected the face from the feature map by extracting image pyramids of various sizes. It has been implemented and available in OpenCV library [22] recently.

**Figure 2:**
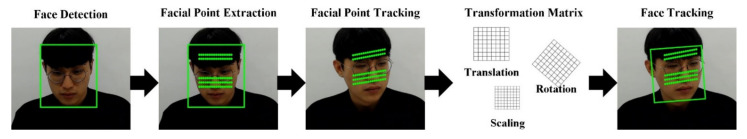
Procedure of face detection and tracking.

In addition, face tracking is important to extract the same facial region from successive frames. The facial region was divided into sub-regions by cropping the middle 50% of the width and top 20% of the height (i.e., forehead) and the middle 50% of the width and middle 25% of height (i.e., nose). Then, 80 facial points were determined within the sub-regions by dividing the forehead region into 32 cells and the nose region into 48 cells, respectively. The facial points were tracked on xy-coordinates by the Kanade-Lucas-Tomasi (KLT) tracker [23]. By empirically considering tracking accuracy and computing cost, the window size of the small subarea cells was determined as the detected face size divided by 10. If the tracked facial points suddenly moves more than 10 pixels, the facial points were re-defined in the forehead and nose regions as described above. Then, the similarity transformation matrix was extracted from locations of the facial points on the previous and next frames to estimate the transformation of the facial region. The similarity transformation matrix hypothesized three characteristics (i.e., translation, rotation, and scaling). Finally, the facial region on next frame was tracked from previous frame by the similarity transformation matrix. Figure 2 shows the procedure of face detection and tracking.

### 2.2. Photoplethysmographic Signal Extraction

**Figure 3:**
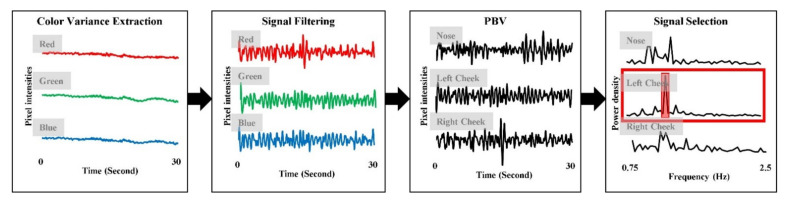
Procedure of photoplethysmographic signal extraction.

Color variation caused by the heartbeat was prominent in the cheek area [24], so that the photoplethysmographic signal was extracted by the RGB spectrums on the middle 50% of the width and middle 25% of height (i.e., nose), on the left 20–35% of width and top 45–70% of height (i.e., left cheek), 220–35% of width and top 45–50% of height (i.e., right cheek). Each RGB signal was normalized by subtracting its mean since the mean indicates melanin components (i.e., skin color) [25]. Then, RBG signals were combined with each other based on pulse blood-volume vector (PBV) modeling [26] for each face area. The combined signals were filtered by a second order Butterworth bandpass filter with a cut-off of 0.75–2.5 Hz corresponding to 45–150 bpm. Finally, the photoplethysmographic signal was extracted by applying the ICA on the filtered signals and selecting the signal with the highest signal to noise ratio (SNR) from three components. The SNR was calculated from the frequency domain of each component as:

*SNR* = *max*(*PS*)/(*∑PS* − *max*(*PS*)), (1)

where *SNR* is a signal to noise ratio and *PS* is a power spectrum of the signal. Figure 3 depicts the procedure of photoplethysmographic signal extraction.

### 2.3. Ballistocardiographic Signal Extraction

**Figure 4:**
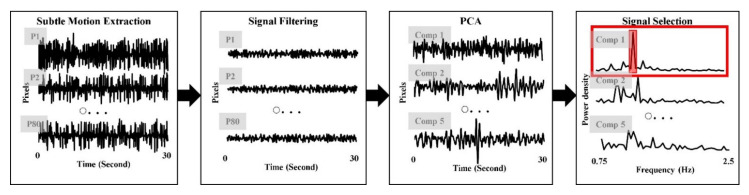
Procedure of ballistocardiographic signal extraction.

Ballistocardiographic head movements were generated up and down by the heartbeat so that the ballistocardiographic signal was extracted by the y-coordinate of each facial point on successive frames. In this study, the 80 ballistocardiographic signals were extracted from the 80 facial points and were normalized by subtracting its mean to make the unit the same as RPPG. The normalized signals were filtered by a second order Butterworth bandpass filter with a cut-off of 0.75–2.5 Hz. Voluntary head movements distorted the signals to have a large amplitude, so that the signals were corrected as the mean if the amplitude is larger than twice the standard deviation. Also, facial expressions distorted the signals extracted from specific muscles (e.g., smile moves the cheek and eye muscles). Thus, the SNR was calculated from each signal and the signals which have lower SNR than mean SNR were removed in the next step. Finally, the ballistocardiographic signal was extracted by applying the PCA on the filtered signals and selecting the signal with the highest SNR from five components. Figure 4 shows the procedure of ballistocardiographic signal extraction.

### 2.4. Signial Combining

**Figure 5:**
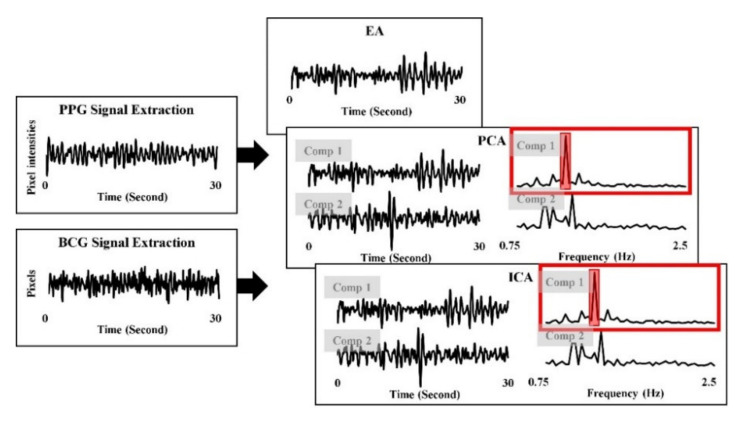
Procedure of signal combining.

This study hypothesized that RPPG are robust to the motion artifacts and sensitive to the illumination variance, whereas RBCG are robust to the illumination variance and sensitive to the motion artifacts. Thus, the photoplethysmographic signal and the ballistocardiographic signal had combined with each other to improve robustness to both the illumination variance and the motion artifacts. This study tested three BSS methods (i.e., EA, PCA, and ICA) to combine RPPG and RBCG. EA was hypothesized to reduce the random noise by averaging repetitive signals and has verified its enhancement as a fusion method in a previous BCG study [27]. EA was calculated as:

*EA* = (*RPPG* + *RBCG*)/*2*, (2)

where *RPPG* is a signal extracted by *RPPG* and *RBCG* is a signal extracted by *RBCG*. In addition, PCA and ICA are the representative BSS methods to reduce the various noise. PCA and ICA were applied on PPG signal and BCG signal extracted from Section 2.2 and Section 2.3 and two components were extracted because the BSS applied on two signals. Then, the combined signal was selected from two components by highest SNR, respectively. Figure 5 depicts the procedure of signal combining.

### 2.5. Heart Rate Estimation

The heart rate estimated from the frequency domain of the signal. The power spectrum was converted from the combined signal by fast Fourier transform (FFT). This study extracted the band of power spectrum between the ranges of 0.75 and 2.5 Hz corresponding to the ranges of 45 and 150 bpm. The dominant frequency was identified with the highest power from the band of power spectrum. The heart rate was calculated by multiplying by the dominant frequency and 60 as:

*HR* = *60* × *freq*, (3)

where *HR* is a heart rate and *freq* is a dominant frequency. The heart rate for RPPG (i.e., $HR_{RPPG}$) was estimated from the PPG signal extracted by Section 2.2, whereas the heart rate for RBCG (i.e., $HR_{RBCG}$) was estimated from the BCG signal extracted by Section 2.3. The heart rates for proposed methods (i.e., $HR_{EA}$, $HR_{PCA}\;and\;HR_{ICA}$) were estimated from the combined signals based on RPPG and RBCG extracted Section 2.4.

## 3. Experiments

The experiments were conducted to evaluate the proposed method according to three measurement conditions in this study. The measurement conditions were determined by illumination and motion artifacts (i.e., normal, facial expressions, and human computer interactions). The experimental procedure was approved by the Institutional Review Board of the Sangmyung University, Seoul, Korea (BE2018-35).

### 3.1. Experiment 1: Normal

This experiment is to collect the normal dataset without illumination variance and motion artifacts. The participants consisted of 20 persons (12 males) and were asked to sit 1 m away from a camera for 3 min with a stationary state. The facial video was recorded by an RGB webcam (Logitech Webcam C270) with 640 × 360 resolution at 30 fps. Also, the ECG signal was simultaneously measured by an ECG measurement system with Lead-I (BIOPAC Systems Inc., Goleta, CA, USA) at a sampling rate of 500 Hz. It was employed as a ground-truth for the evaluation of the proposed methods.

### 3.2. Experiment 2: Facial Expressions

This experiment is to collect the dataset with facial expressions. The 20 persons who participated in experiment 1 were also asked to sit in front of a camera for 3 min with a stationary state. They then followed the six basic facial expressions (i.e., happiness, sadness, surprise, anger, disgust, and fear), which were displayed on the monitor for 30 s in random order to minimize the ordering effect. The facial video and the ECG signal were recorded and measured at the same manner in experiment 1.

### 3.3. Experiment 3: Human Computer Interactions

This experiment is to collect the dataset including illumination variance and motion artifacts occurred in human computer interactions. The 17 persons (8 males) were participated and asked to watch the four videos to cause to some emotions. Then, they wrote their emotions on self-report by two-dimensional model [28]. While watching the videos and writing the self-report, they asked to freely express the facial expressions and move their head. Also, the illumination on the face was changed by reflecting the light from the video. The facial video was recorded by an RGB webcam (Logitech Webcam C270) with a 1920 × 1080 resolution at 30 fps. The ECG signal was also measured at the same manner in experiment 1.

## 4. Results

This study demonstrated the improvement of proposed fusion methods by comparing them with previous RPPG and RBCG methods. The heart rate of ECG was calculated by the QRS detection algorithm [29] and determined as the ground-truth for evaluation of the proposed methods. The proposed method was verified by calculating the four metrics and by representing the one plot as follows: mean absolute error (MAE), standard deviation of absolute error (SDAE), root mean squared error (RMSE), Pearson’s correlation coefficient (CC), and the Bland-Altman plot. MAE, SDAE, and RMSE describes the difference of mean heart rate and variation, respectively. CC determines the statistical similarity of heart rates over time, so that the coefficient value indicates a strong positive similarity if it is approaching to the one. The Bland-Altman plot represents graphically the statistical differences by assigning the mean (x-axis) and difference (y-axis) between the two measurements. The line on the plot is indicated the 95% limits of an agreement based on mean difference and the ±1.96 standard deviation of the differences. The statistical parameters were calculated from the heart rates for RPPG ($i.e.,\;HR_{RPPG}$), RBCG ($i.e.,\;HR_{RPPG}$), and the proposed methods (i.e., $HR_{EA}$, $HR_{PCA}\;and\;HR_{ICA}$) by comparing them with ECG.

### 4.1. Experiment 1: Normal

Table 1 shows the estimation of heart rates from the normal dataset using RPPG, RBCG, and the proposed fusion methods (i.e., EA, PCA, and ICA). All fusion methods were more accurate than RPPG and RBCG. In addition, the ICA-based fusion method showed the lowest errors in the normal dataset (MAE = 1.04, SDAE = 0.91, RMSE = 1.39, CC = 0.999).

**Figure 6:**
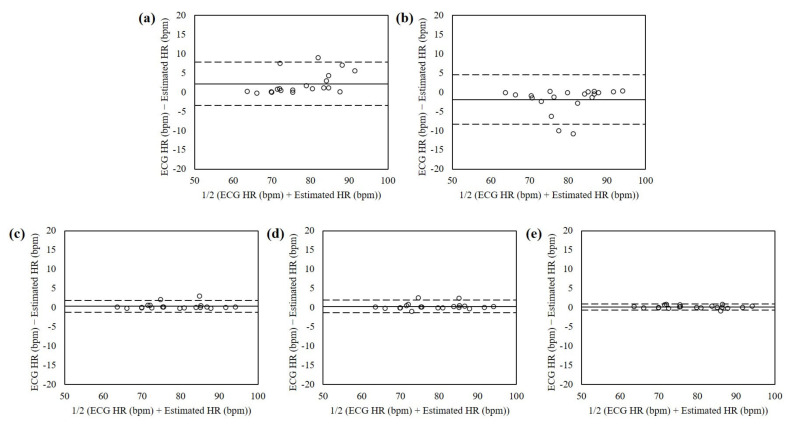
Bland-Altman plots of heart rates estimated from the normal dataset without illumination variance and motion artifacts using interactions using (**a**) RPPG, (**b**) RBCG, (**c**) EA, (**d**) PCA, and (**e**) ICA. The lines are the mean errors and 95% LOA.

The Bland-Altman plots of estimated heart rates from the normal dataset without illumination variance and motion artifacts using RPPG, RBCG, and the proposed fusion methods are shown in Figure 6. The mean errors were 2.19 with 95% limits of agreement (LOA) in −3.43 to 7.81 (RPPG), −1.93 with 95% LOA in −8.35 to 4.49 (RBCG), 0.37 with 95% LOA in −1.19 to 1.93 (EA), 0.34 with 95% LOA in −1.26 to 1.94 (PCA), and 0.18 with 95% LOA in −0.63 to 0.98 (ICA). The heart rates estimated using the ICA-based fusion method showed the lowest mean difference and variances.

### 4.2. Experiment 2: Facial Expressions

The heart rates were estimated from the dataset with facial expressions using RPPG, RBCG, and the proposed fusion methods as shown in Table 2. All fusion methods showed lower errors than RPPG and RBCG. In addition, the errors of the PCA-based fusion method were lower than one of other methods (MAE = 2.76, SDAE = 2.34, RMSE = 3.23, CC = 0.968).

**Figure 7:**
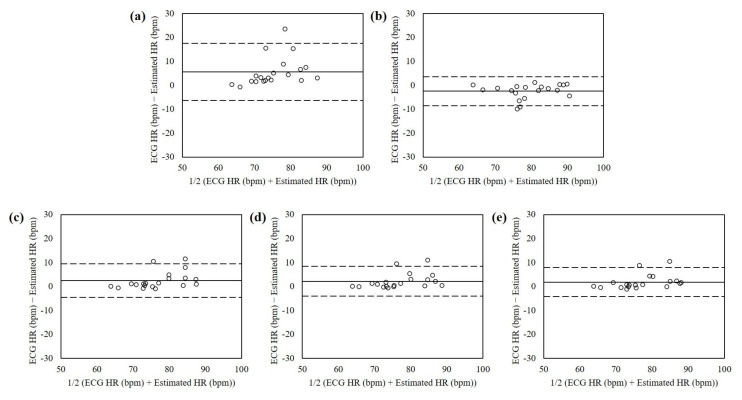
Bland-Altman plots of heart rates estimated from the dataset with facial ex-pressions interactions using (**a**) RPPG, (**b**) RBCG, (**c**) EA, (**d**) PCA, and (**e**) ICA. The lines are the mean errors and 95% LOA.

Figure 7 shows the Bland-Altman plots of the heart rates estimated from the dataset with facial expressions using RPPG, RBCG, and the proposed fusion methods. The mean errors were 5.54 with 95% limits of agreement (LOA) in −6.39 to 17.48 (RPPG), −2.48 with 95% LOA in −8.56 to 3.59 (RBCG), 2.48 with 95% LOA in −4.59 to 9.55 (EA), 2.20 with 95% LOA in −4.04 to 8.45 (PCA), and 1.87 with 95% LOA in −4.12 to 7.86 (ICA). The heart rates estimated using the PCA-based fusion method showed the lowest mean difference and variances.

### 4.3. Experiment 3: Human Computer Interactions

Table 3 shows the estimation of heart rates from the dataset in human computer interactions using RPPG, RBCG, and the proposed fusion methods. All fusion methods showed lower errors than RPPG and RBCG. Unlike the other datasets, the EA-based fusion method showed lowest errors in this dataset (MAE = 4.79, SDAE = 2.13, RMSE = 5.181, CC = 0.629).

**Figure 8:**
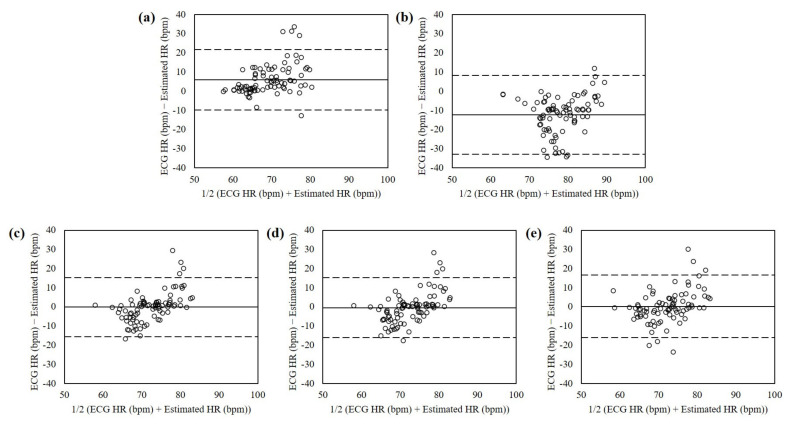
Bland-Altman plots of heart rates estimated from the dataset in human computer interactions using (**a**) RPPG, (**b**) RBCG, (**c**) EA, (**d**) PCA, and (**e**) ICA. The lines are the mean errors and 95% LOA.

Figure 8 shows the Bland-Altman plots of the heart rates estimated from the dataset in human computer interactions using RPPG, RBCG, and the proposed fusion methods. The mean errors were 6.05 with 95% limits of agreement (LOA) in −9.71 to 21.81 (RPPG), −12.35 with 95% LOA in −32.88 to 8.18 (RBCG), −0.09 with 95% LOA in −15.49 to 15.32 (EA), −0.49 with 95% LOA in −15.14 to 15.16 (PCA), and 0.25 with 95% LOA in −16.15 to 16.65 (ICA). The heart rates estimated using the EA-based fusion method showed the lowest mean difference and variances.

## 5. Discussion

In this study, the fusion method based on RPPG and RBCG was developed to enhance the heart rate estimation using EA, PCA, and ICA. This study evaluated the proposed method on three datasets according to illumination variance and motion artifacts as follows: (1) normal, (2) facial expressions, and (3) human computer interactions. The proposed method was more accurate than the previous RPPG and RBCG in all datasets. This result indicated that with the advancement of fusion methods based on RPPG and RBCG, ECG could eventually be replaced by remote sensing in daily life. Thus, this study strongly encourages the fusion method based on RPPG and RBCG as a requirement to estimate heart rate using a camera more accurately.

Overall, this study has drawn four significant findings. First, PCA and ICA were better than EA in the datasets including less noise such as normal and facial expressions. It indicated that the BSS algorithms can be improved if RPPG and RBCG are enhanced by reducing the noise. On the other hand, EA was better than PCA and ICA in daily life not yet.

Second, experiment 3 evaluated the proposed method and the previous RPPG and RBCG in human computer interactions environment which is similar to daily life. As shown in the Bland-Altman plots (Figure 7), the previous RPPG and RBCG estimated the heart rate higher (LOA in −9.71 to 21.81) and lower (LOA in −32.88 to 8.18) than the ground-truth (i.e., ECG), respectively. It indicated that RPPG has high frequency noise whereas RBCG has low frequency noise. The illumination variance has high frequency because it appears at a high rate that humans cannot perceive. On the other hand, the motion artifacts have low frequency because they occur instantaneously and briefly. RPPG and RBCG are sensitive to the illumination variance and motion artifacts, respectively, so that the result is acceptable. Note that the proposed method reduced both illumination variance and motion artifacts (LOA in −15.49 to 15.32) since it has low frequency and high frequency noise evenly and little.

Third, this study showed significant results compared to other fusion methods. Particularly, Liu et al. [13] developed a fusion method, combined RPPG with BCG measured using an additional motion sensor, and showed MAE of 6.20 bpm in motion state. Although the measurement condition is different, our proposed method showed MAE of 4.79 bpm in experiment 3 including illumination variance and motion artifacts. Note that it indicates possibility of combining PPG and BCG with only a camera without an additional sensor.

Finally, this study presented the fusion method based on RPPG and RBCG to enhance the heart rate estimation using a camera. Although it should be more improved, a novel approach was developed for the possibility of practical use of remote sensing in daily life. Many studies have been presented before, but it has not been developed as a product for real users due to restrictions on the use environment. The product for real users should be developed and tested to minimize the measurement burden and to improve the use environment, so that it can be used in practical domains such as self-driving cars or non-face-to-face communication.

## 6. Conclusions

This study developed a fusion method to estimate heart rate from facial videos based on RPPG and RBCG. The proposed methods using EA, PCA, and ICA had compared them with the previous RPPG and RBCG. As a result, the proposed methods showed enhanced accuracy from three datasets according to illumination variance and motion artifacts. The findings are a significant step toward ensuring the enhanced development of RPPG and RBCG. This study is expected to contribute to enhanced heart rate measurement by overcoming noise of illumination variance and motion artifacts and consequently improve the possibility of applications of remote sensing in daily life.

## Figures and Tables

**Table 1:** Estimation of heart rates from the normal dataset without illumination variance and motion artifacts.

Methods	MAE (bpm)	SDAE (bpm)	RMSE (bpm)	CC (r)
RPPG	1.84	1.66	2.49	0.981 **
RBCG	2.56	2.26	3.48	0.927 **
Fusion (EA)	1.05	0.93	1.42	0.996 **
Fusion (PCA)	1.08	0.99	1.49	0.996 **
Fusion (ICA)	**1.04**	**0.91**	**1.39**	0.999 **

MAE, mean absolute error; SDAE, standard deviation of absolute error; RMSE, root mean square error; CC, Pearson’s correlation coefficient. Two asterisk represents significant correlation levels at *p*-value < 0.01. The lowest error and highest correlation values are bolded.

**Table 2:** Estimation of heart rates from the dataset with facial expressions.

Methods	MAE (bpm)	SDAE (bpm)	RMSE (bpm)	CC (r)
RPPG	3.13	2.59	4.09	0.947 **
RBCG	3.94	4.07	5.71	0.920 **
Fusion (EA)	3.06	2.56	4.53	0.955 **
Fusion (PCA)	**2.76**	**2.34**	**3.23**	0.968 **
Fusion (ICA)	2.99	2.53	4.17	**0.972** **

MAE, mean absolute error; SDAE, standard deviation of absolute error; RMSE, root mean square error; CC, Pearson’s correlation coefficient. Two asterisk represents significant correlation levels at *p*-value < 0.01. The lowest error and highest correlation values are bolded.

**Table 3:** Estimation of heart rates from the dataset in human computer interactions.

Methods	MAE (bpm)	SDAE (bpm)	RMSE (bpm)	CC (r)
RPPG	5.68	2.93	6.53	0.713 **
RBCG	14.06	6.75	15.86	0.051
Fusion (EA)	**4.79**	**2.13**	**5.81**	0.629 **
Fusion (PCA)	5.42	4.2.84	6.13	0.622 **
Fusion (ICA)	5.66	3.59	6.48	0.617 **

MAE, mean absolute error; SDAE, standard deviation of absolute error; RMSE, root mean square error; CC, Pearson’s correlation coefficient. Two asterisk represents significant correlation levels at *p*-value < 0.01. The lowest error and highest correlation values are bolded.

## Data Availability

The data presented in this study are available on request from the corresponding author. The data are not publicly available due to privacy.

## References

[1] Rouast P.V., Adam M.T.P., Chiong R., Cornforth D., Lux E. (2018). Remote heart rate measurement using low-cost RGB face video: A technical literature review. Front. Comput. Sci..

[2] Hertzman A.B., Dillon J.B. (1940). Applications of photoelectric plethysmography in peripheral vascular disease. Am. Heart J..

[3] Kamshilin A.A., Nippolainen E., Sidorov I.S., Vasilev P.V., Erofeev N.P., Podolian N.P., Romashko R.V. (2015). A new look at the essence of the imaging photoplethysmography. Sci. Rep..

[4] Poh M.Z., McDuff D.J., Picard R.W. (2010). Non-contact, automated cardiac pulse measurements using video imaging and blind source separation. Opt. Express.

[5] Xu L., Cheng J., Chen X. (2017). Illumination variation interference suppression in remote PPG using PLS and MEMD. Electron. Lett..

[6] Zhang Y., Dong Z., Zhang K., Shu S., Lu F., Chen J. (2021). Illumination variation-resistant video-based heart rate monitoring using LAB color space. Opt. Lasers Eng..

[7] Starr I., Rawson A.J., Schroeder H.A., Joseph N.R. (1939). Studies on the Estimation of Cardiac Output in Man, and of Abnormalities in Cardiac Function, from the heart’s Recoil and the blood’s Impacts; the Ballistocardiogram. Am. J. Physiol. Leg. Content.

[8] Balakrishnan G., Durand F., Guttag J. Detecting pulse from head motions in video. Proceedings of the IEEE Conference on Computer Vision and Pattern Recognition (CVPR).

[9] Shan L., Yu M. Video-based heart rate measurement using head motion tracking and ICA. Proceedings of the 2013 6th International Congress on Image and Signal Processing (CISP).

[10] Haque M.A., Nasrollahi K., Moeslund T.B., Irani R. (2016). Facial video-based detection of physical fatigue for maximal muscle activity. IET Comput. Vis..

[11] Hassan M.A., Malik A.S., Fofi D., Saad N.M., Ali Y.S., Meriaudeau F. (2017). Video-Based Heartbeat Rate Measuring Method Using Ballistocardiography. IEEE Sens. J..

[12] Shao D., Tsow F., Liu C., Yang Y., Tao N. (2016). Simultaneous monitoring of ballistocardiogram and photoplethysmogram using a camera. IEEE Trans. Biomed. Eng..

[13] Liu Y., Qin B., Li R., Li X., Huang A., Liu H., Liu M. (2021). Motion-Robust Multimodal Heart Rate Estimation Using BCG Fused Remote-PPG With Deep Facial ROI Tracker and Pose Constrained Kalman Filter. IEEE Trans. Instrum. Meas..

[14] Viola P., Jones M. Rapid object detection using a boosted cascade of simple features. Proceedings of the IEEE Conference on Computer Vision and Pattern Recognition (CVPR).

[15] OpenCV: Cascade Classifier. https://docs.opencv.org/4.2.0/db/d28/tutorial_cascade_classifier.html.

[16] Dalal N., Triggs B. Histograms of oriented gradients for human detection. Proceedings of the IEEE Computer Society Conference on Computer Vision and Pattern Recognition (CVPR).

[17] Dlib: Face Detector. http://dlib.net/face_detector.py.html.

[18] Abadi M., Barham P., Chen J., Chen Z., Davis J., Kudlur M. Tensorflow: A system for large-scale machine learning. Proceedings of the USENIX Symposium on Operating Systems Design and Implementation (OSDI).

[19] Liu W., Anguelov D., Erhan D., Szegedy C., Reed S., Fu C.Y., Berg A.C. Ssd: Single shot multibox detector. Proceedings of the European Conference on Computer Vision (ECCV).

[20] He K., Zhang X., Ren S., Sun J. Deep residual learning for image recognition. Proceedings of the IEEE Conference on Computer Vision and Pattern Recognition (CVPR).

[21] Yang S., Luo P., Loy C.C., Tang X. Wider face: A face detection benchmark. Proceedings of the IEEE Conference on Computer Vision and Pattern Recognition (CVPR).

[22] OpenCV: Face Detector by SSD in DNN Module. https://github.com/opencv/opencv/tree/master/samples/dnn/face_detector.

[23] Bouguet J.Y. (2001). Pyramidal implementation of the affine lucas kanade feature tracker description of the algorithm. Intel Corp..

[24] Lempe G., Zaunseder S., Wirthgen T., Zipser S., Malberg H. ROI selection for remote photoplethysmography. Proceedings of the Bildverarbeitung für die Medizin.

[25] De Hann G., Jeanne V. (2013). Robust pulse rate from chrominance-based rPPG. IEEE Trans. Biomed. Eng..

[26] De Haan G., Van Leest A. (2014). Improved motion robustness of remote-PPG by using the blood volume pulse signature. Physiol. Meas..

[27] Lee H., Lee H., Whang M. (2018). An enhanced method to estimate heart rate from seismocardiography via ensemble averaging of boy movements at six degrees of freedom. Sensors.

[28] Russell J.A. (1980). A circumplex model of affect. J. Personal. Soc. Psychol..

[29] Pan J., Tompkins W.J. (1985). A real-time QRS detection algorithm. IEEE Trans. Biomed. Eng..

